# The Influence of the Use of Pyrolysis Oil as a Binder on the Physicochemical Properties of Pellets

**DOI:** 10.3390/ma18132935

**Published:** 2025-06-20

**Authors:** Bryan Romankiewicz, Błażej Gaze

**Affiliations:** 1 Faculty of Life Sciences and Technology, Wroclaw University of Environmental and Life Sciences, 50-363 Wroclaw, Poland; 120354@student.upwr.edu.pl; 2 Institute of Agricultural Engineering, Wroclaw University of Environmental and Life Sciences, 51-630 Wroclaw, Poland

**Keywords:** agricultural waste, pyrolysis oil, formed fuels, emission reduction, biomass

## Abstract

The article presents the results of research on the effect of pyrolysis oil used as a binder in the pelletization process. The materials used to produce pyrolysis bio-oil were municipal organic waste and residues from greenhouse tomato production. The research assessed the mechanical strength, physicochemical properties, and modifications of the energy and emission parameters of the produced pellets. As a result, formed fuels were obtained, whose physicochemical properties, among others, were improved in terms of combustion heat (the value increased by up to 15.7%). After selected binders were used, the mechanical strength of the fuels also increased, which in the best variant increased by 2.87%. In all research cycles, valuable data was obtained that can be used, for example, in companies producing formed fuels, as well as in the agri-food industry, where a large amount of waste is generated, the properties of which have not previously allowed their use for energy purposes.

## 1. Introduction

Fossil fuels continue to play a key role in meeting the global energy demand [1]. The expected population growth, as well as the constantly growing demand for energy caused by increasingly energy-intensive lifestyles, will certainly contribute to an increase in their consumption in the coming years [1,2].

Unfortunately, the use of these fuels has many major disadvantages. Fossil fuels are non-renewable, and their resources may be exhausted soon [3]. Another disadvantage of fossil fuels is the enormous emissions of greenhouse gases, which in the long run may have disastrous consequences for human health, life, and the environment [4].

The excessive use of gas and crude oil significantly increases carbon dioxide emissions, which leads to an increase in global temperature. The increase in average global temperature has a significant negative impact on the global and local climate. It also has a negative impact on human health and the agricultural sector, which translates into the stability of national economies [4,5].

As climate change is a multidimensional challenge affecting virtually every aspect of life, the European Union has decided to implement the “Fit for 55” package, which is a set of strategies and instruments aimed at reducing greenhouse gas emissions by 55% by 2030 and achieving net zero emissions by 2050 [6].

To meet our climate goals and reduce the risks posed by the continued use of fossil fuels, we need to focus on finding more stable and renewable fuels. A promising alternative to fossil fuels is renewable energy sources such as wind and solar. However, they do not allow the production of gaseous or liquid fuels for the transport sector or the production of electricity and heat in the cogeneration process. Moreover, their availability is seasonal and depends largely on weather and environmental conditions [7].

A stable alternative to the above-mentioned sources is biomass, which can be transformed into biofuels and used to produce energy regardless of the season or location [8]. Biomass energy is particularly valuable because it is available in most regions of the world. Biomass can be thermally converted to produce heat or electricity or transformed into other energy carriers, such as biogas or liquid biofuels [8].

Thanks to pelletization, biomass can be transformed into high-energy granulates with uniform dimensions and increased bulk density. Energy is also concentrated in a unit of mass, which improves its energy properties and facilitates transport and storage [9]. Biomass pellets are used both in households and in industry, constituting fuel for grate boilers and fluidized bed gasification technology [10]. The increasing popularity of biomass as an energy source has contributed to the dynamic development of the pellet market, which has expanded significantly in recent years and is expected to continue to grow rapidly [10].

Biomass is currently recognized as the third largest global energy source. In developing countries, where agricultural and forest areas dominate, biomass constitutes 40–50% of the media used to produce energy and heat. Green plants produce biomass through photosynthesis, converting sunlight into organic matter [6].

Biomass conversion processes include thermochemical methods, such as gasification, pyrolysis, and combustion, and biological methods, such as fermentation [11].

One of the increasingly popular biomass processing processes is pyrolysis, which enables the effective extraction of fuels and raw materials [11]. Pyrolysis is a process that involves transforming materials by converting energy into another form. These changes take place at high temperatures and in the absence of oxygen in the reactor [12]. As a result of pyrolysis from biomass, three basic products are obtained: biochar, bio-oil, and pyrolysis gas [12].

Bio-oil is a dark brown liquid with very high oxygenation [13]. It is a thick, condensed mixture with an intense, characteristic odor and an elemental composition like biomass. It contains numerous oxygen compounds and a significant amount of water, originating from both biomass moisture and chemical reactions occurring during its formation [13]. It may contain carbon particles and dissolved alkali metals from the ash, which additionally affects its properties [13]. Its final composition is strongly determined by the type of biomass, process conditions, technology used, and the efficiency of carbon separation and condensation [13].

The use of bio-oil as a binder fits into the assumptions of the circular economy and the Zero Waste concept [14]. A key element of effective waste management is the protection of available secondary resources through a responsible approach to the production, consumption, reuse, and recovery of raw materials [14,15]. Zero Waste principles emphasize effective material management and waste reduction [15].

The European Union produces almost 17 million tons of tomatoes annually, of which Poland is responsible for approximately 920 thousand tons, which places it in eighth place among European producers [6]. It is estimated that around 3 million tons of tomato processing waste are generated across the EU, with the amount depending on the production methods used [16].

Such a large scale of both cultivation and waste creates the need for effective solutions for their management. In the face of the growing importance of biomass in energy strategies, the appropriate use of these residues may be an important element of sustainable raw material management [16].

Considering the growing pressure to use organic post-production residues and the desire to improve the quality of formed biomass fuels, research was undertaken to assess the impact of production and use of pyrolytic bio-oils as a binder in pelletization processes. Literature research and information coming from the biofuel processing and production sector have revealed a research gap in this area. As part of the work carried out, the quality of the obtained products, their physicochemical properties, and the impact of the use of bio-oil on the energy and emission parameters of biofuels were analyzed.

## 2. Materials and Methods

### 2.1. Research Stand

The tests were carried out in a low-power, fully automatic biomass boiler with a nominal power of 60 kW (Biowarmer 60, manufactured by Cichewicz kotły c.o., Rzemieślnicza 11 street, 09-100 Płońsk, Poland). The device’s operating parameters were regulated using a controller. The temperature sensor and lambda sensor readings allowed the controller to select the appropriate dose of fuel and air supplied to the combustion chamber. The exhaust fan and screw feeder coupled with the pellet tank provided the appropriate dose of air and fuel. The heat generated in the process of thermal conversion of fuel was transferred to the environment using three fan heaters with a heating power of 30 kW each. Basic technical information about the parameters of the pellet boiler used is presented in Table 1.

The pyrolysis process of waste materials was carried out using a low-power pyrolysis reactor (produced by Büchiglasuster, Uster, Switzerland). The technical specifications of this device can be found in Table 2.

The pellets were produced using a pellet production line with a power of 11 kW (Kovo Novak production line, model MGL 200 by Kovo Novák, Citonice, Czech Republic). The production process was carried out using a die with a mesh diameter of 6 mm, which allowed pellets to be obtained with a diameter of 6 mm and a length of approximately 20–30 mm.

### 2.2. Materials

**Figure 1:**
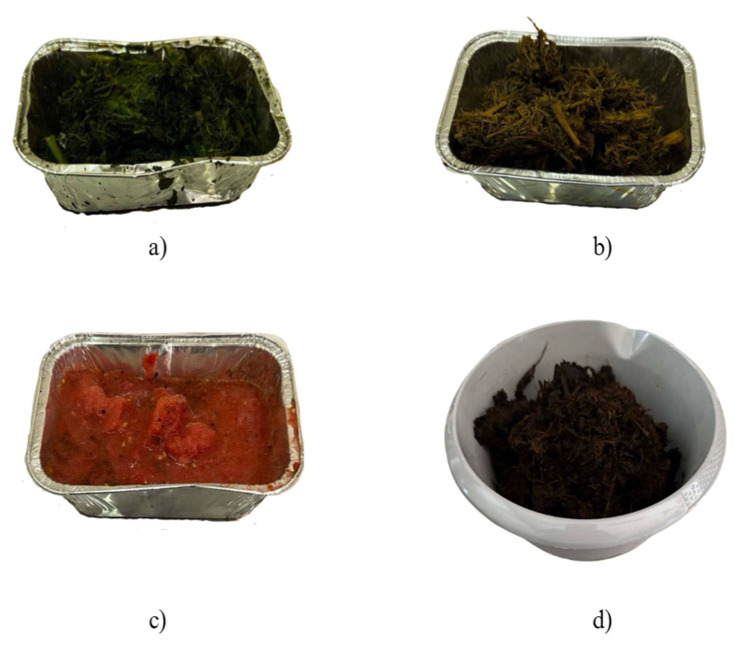
Substrates used to produce BIO oil: (**a**) fresh haulms of tomatoes, (**b**) dry haulms of tomatoes, (**c**) tomato fruits, and (**d**) bio-waste.

Four types of biomasses were used to produce bio-oil: post-production residues from tomato production and processing (dry tomato haulms (ad. 1), fresh tomato haulms (ad. 2), tomato fruits (ad. 3)), and selectively collected bio-waste (ad. 4). The substrates are presented in the figures (Figure 1).

Tomato haulms and fruits came from a local company dealing with tomato cultivation and processing (Citronex, Siechnice, Poland) and bio-waste from a waste management plant (Łużyckie Centrum Recyklingu, Marszów, Poland).

Molded fuels, in this case pellets with a diameter of 6 mm and a length of 30 mm, were made from wood dust remaining after processing deciduous trees (oak and acacia), obtained from a local sawmill (Zwyż-Dźwig s. c, Żagań, Poland). The material came mainly from carpentry grinding and cutting processes, which resulted in its high homogeneity and low humidity. The particle fraction was less than 500 µm, which ensured good forming properties. Particle size was determined by manual sieving using a 500 µm sieve.

Pyrolysis oils were produced by low-temperature pyrolysis (torification) under controlled conditions of a temperature of 200 °C and atmospheric pressure using a pressure reactor (produced by Büchiglasuster, Uster, Switzerland). Before thermal treatment, each raw material was ground in a laboratory grinder to a fraction smaller than 500 µm.

Before the start of each torrefaction cycle, a reactor leak test was performed for one minute. After its positive completion, the biomass sample was placed in the reactor and the heating mantle was turned on. The pyrolysis process lasted 60 min after reaching the set temperature. After the process, the pyrolysis gas, the resulting solid part (biochar), and the bio-oil were separated.

**Figure 2:**
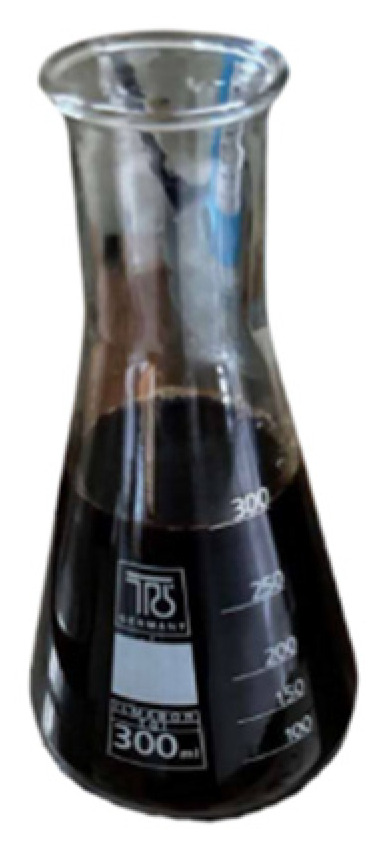
Bio-oil produced in the pyrolysis process.

As a result of thermal treatment, three product fractions were obtained: torreficate accounted for 40%, pyrolysis oil 11%, and pyrolysis gas 49% of the total. The drawing below (Figure 2) shows an example of bio-oil produced in the pyrolysis process. 

After lowering the sample temperature, the liquid fraction was separated from the solid fraction as a result of the filtration and sedimentation process. After cooling the material, the settled solid fraction, thanks to its higher density, gradually began to sink to the bottom of the vessel, while the liquid fraction, with a lower density, remained on the surface. The sedimentation time was approximately 30 min, which ensured the effective separation of both fractions. To speed up this process, fine-porosity filters were used, which made it possible to remove even the smallest solid particles, leaving a clean liquid fraction.

### 2.3. The Process of Producing Molded Fuels

Molded fuels were produced from wood biomass by the appropriate preparation of the raw material, shaping, and stabilization of the product. A key aspect of the process was the deliberate addition of water and pyrolysis oil, which influenced the final properties of the fuel. The dust was pre-homogenized before further processing.

To ensure the appropriate plasticity of the mixture, the humidity was increased to 20–25%. After preparing the wood dust, 7% (0.7 kg) of previously produced pyrolysis oil was added to the material, which acted as a binder and constituted a source of additional energy in the finished fuel. Mixing took place in a vertical mixer with a screw mixer, which ensured the uniform distribution of the oil throughout the raw material. The mixing time was 15 min, and the mixer speed was 50 rpm. Thanks to the mixing process, the material obtained a homogeneous consistency, and the dust particles were effectively combined, which facilitated further fuel formation. At the same time, under the same conditions, a batch of pellets was produced in which no binder was added. In this way, 5 batches of material ready for the forming process were prepared. The prepared mixture was pelletized, which resulted in a thickening of the structure and a reduction in humidity to 8–10%. After pelletizing, the formed fuels were left to cool, which allowed their structures to stabilize and excess moisture to evaporate.

Ready-made fuels were stored in tight containers to minimize their degradation and maintain appropriate energy parameters.

### 2.4. Biofuel Physicochemical Analysis

The heat of combustion of raw molded fuels was determined in accordance with the standard [17] using the IKA C 5010 calorimeter (IKA-Werke GmbH & Co. KG, Staufen, Germany). The measurement was carried out for samples weighing approximately 1 g, each time in three repetitions. The technical specifications of the IKA C 200 calorimeter are shown in Table 3.

The content of volatile matter, ash (total mineral residues), and moisture was determined in accordance with applicable standards [18,19,20] using the LECO TGA 701 thermogravimetric analyzer (LECO Corporation, St. Joseph, MI, USA) (its technical data is presented in Table 4). Analytical samples, ground to fractions below 500 µm, were weighed in the range of 0.8–1.2 g. Each analysis was performed in triplicate.

Elemental composition of biomass materials, i.e., the determination of carbon, hydrogen, nitrogen, and sulfur content, was performed using the PerkinElmer CHNS/O 2400 analyzer (PerkinElmer, Inc., Waltham, MA, USA). The analysis was performed on dry samples fragmented to a particle size below 0.2 mm, following the PN-EN standard [21]. The technical data of the used device is contained in Table 5.

The mechanical strength of the pellets was tested in accordance with the standard [22] using a dedicated measuring device. A 1000 g sample was placed in a rotating test chamber and rotated for 10 min at a speed of 50 rpm. After the test was complete, the material was sieved through a 3.15 mm sieve, and the strength coefficient was calculated as the ratio of the initial mass to the mass of the over-sieve fraction.

### 2.5. Measurement of Exhaust Gases Composition

The concentration of carbon monoxide (CO) and nitrogen oxides (NOx) in exhaust gases was measured using the TESTO 350 exhaust gas analyzer (Testo SE & Co. KGaA, Lenzkirch, Germany) in accordance with the standard [23]. The measuring probe was placed on the chimney stub located 40 cm behind the boiler, maintaining a tight connection, which prevented the ingress of outside air. The depth of lance placement was determined based on measurements of the flow and temperature of the exhaust stream. Concentrations were recorded every second for 4 h.

The content of suspended dust in the exhaust gases was measured using a Testo 380 dust meter. Before starting the measurements, the device was conditioned and the measurement probe was heated to a temperature of 120 °C. The measurement was carried out for 4 h in continuous mode, with data recorded every 1 s.

### 2.6. Measurement of the Combustion Process Temperature

The temperature in the combustion chamber was measured following the PN-EN standard. The temperature was registered based on the PAR AR205 recorder (Princeton Applied Research (AMETEK Scientific Instruments, Oak Ridge, TN, USA), to which three K-type thermocouples were connected. The temperature was recorded every second for the entire period of boiler operation. The mean measurement error in the results has been determined at a level of ±1.5 °C.

## 3. Results

A series of tests were carried out to determine and compare the impact of the use of pyrolysis bio-oil used as a binder on the physicochemical properties of the produced pellets. The measured values in individual measurement cycles were averaged and converted into normative values.

To facilitate the analysis of the results, individual binders were marked as follows: bio-oil from dry tomato haulm as ad.1, bio-oil from green tomato haulm as ad.2, bio-oil from tomato fruit as ad.3, and bio-oil from municipal bio-waste as ad.4.

### 3.1. Physicochemical Analysis of Materials

All pellet samples were subjected to physicochemical analysis, including elemental analysis, the results of which are summarized in Table 6.

The analysis of the elemental composition showed that the use of different types of bio-oils resulted in a change in the content of the most important elements in biofuels. Compared to the reference sample, there was a significant increase in the nitrogen and sulfur content in all samples with the addition of binder. The total carbon content, which influences the calorific value, also increased for all modified pellets, reaching up to 55% in the fuel sample with a bio-waste binder. The hydrogen content was maintained at a similar level in all samples (8.2–8.5%).

In addition to the elemental analysis, a technical analysis was carried out, which included determining the calorific value and moisture, volatile matter, and ash (total mineral residues) content. The obtained results are summarized in Table 7.

The use of bio-oil as a binder significantly improved the technical characteristics of the tested pellets. The calorific value in all samples with the addition of bio-oil was higher than in the reference pellet, reaching the maximum value of 23.8 MJ∙kg^−1^ for the fuel with the addition of bio-oil from bio-waste. The content of volatile particles, which influences the ease of ignition and combustion intensity, was the lowest in the pellet with the addition of a binder with dry haulms (6.6%), and the highest in the sample with biowaste (15.7%). It should be noted that despite the overall increase in calorific value, some samples showed higher moisture content, which may negatively affect combustion properties. Higher humidity reduces the effective calorific value of the fuel because part of the energy is used to evaporate water. The ash content remained low in all cases, indicating that the addition of bio-oil as a binder did not significantly affect this parameter and did not increase the amount of ballast in the fuel.

**Figure 3:**
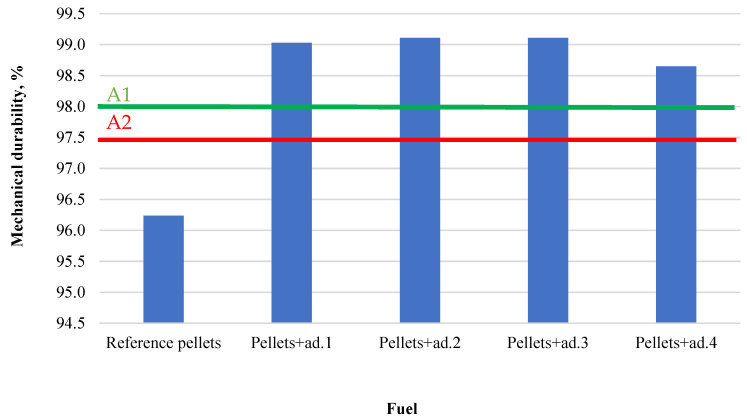
Mechanical strength of pellets.

The mechanical strength results (Figure 3) for the produced pellets are presented in the chart below. 

The addition of bio-oil significantly improved the mechanical resistance of the pellets. The best results were obtained for pellets with the addition of bio-oil from tomato fruit (ad.3, 99.11%), green tomato haulms (ad.2, 99.09%), and dry tomato haulms (ad.1, 99.03%), all of which qualify for the highest A1 quality class according to the standard [21]. The sample with bio-oil from municipal bio-waste (ad.4, 98.65%) also meets A1 requirements, though it demonstrated the lowest strength among the modified pellets. The reference pellet (without binder) achieved a value of 96.24%, falling below the A1 and A2 thresholds. These results confirm that bio-oil binders significantly improve the structural integrity of pellets, though the degree of improvement depends on the biomass source of the oil.

### 3.2. Results of Temperature Distribution in the Combustion Chamber of the Boiler

**Figure 4:**
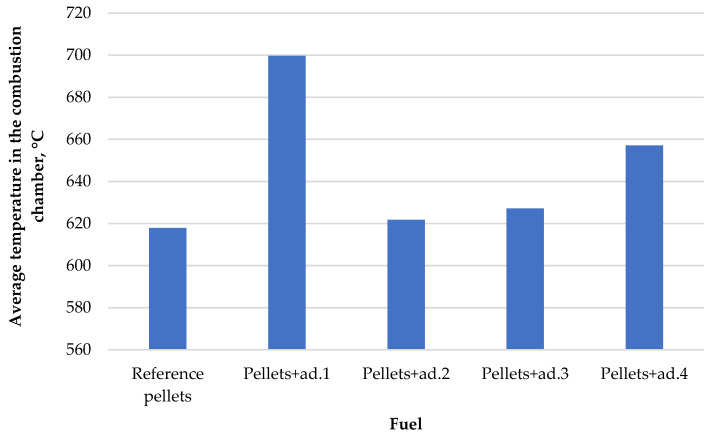
Average temperatures in the combustion chamber.

The chart below (Figure 4) shows a comparison of average temperatures in the combustion chamber for pellets with the addition of various binders. 

The use of different binders significantly influenced the combustion intensity, as reflected by variations in average combustion chamber temperatures. The highest average temperature (699.67 °C) was recorded for pellets with the addition of bio-oil from dry tomato haulms (ad.1), likely due to its favorable chemical composition and high calorific value. The next highest temperatures were observed for ad.4 (municipal bio-waste) and ad.3 (tomato fruit), while ad.2 (green tomato haulms) showed a slightly lower value. The reference sample, without any binder, had the lowest average combustion temperature (617.87 °C), indicating reduced combustion efficiency. These differences highlight the impact of binder origin on combustion behavior.

### 3.3. Analysis of Exhaust Gas Composition

**Figure 5:**
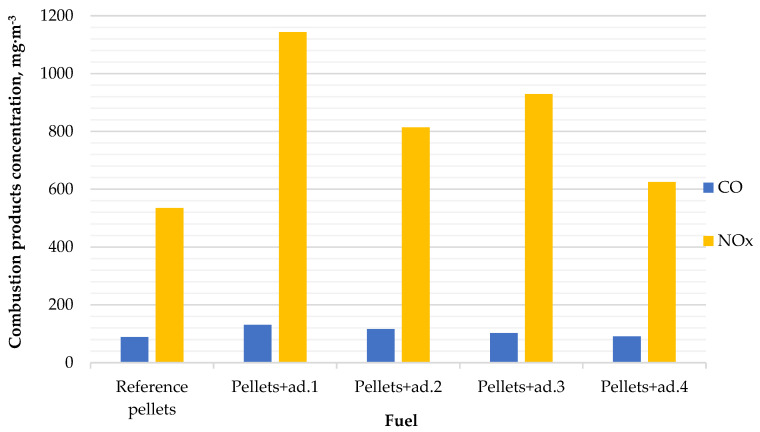
The content of CO and NO_x_ in exhaust gases.

Thanks to the analysis of the exhaust gas composition, it is possible to assess the efficiency of the combustion process and the potential impact on the environment. The most important thing is to determine the emission of carbon oxides (CO) and nitrogen oxides (NO_x_). They are indicators of the intensity of thermal transformations and the quality of combustion. The results of the emission analysis (Figure 5) are presented in the chart below. 

The highest CO emission (131.47 mg∙m^−3^) was recorded for pellets containing bio-oil from dry tomato haulms (ad.1), while the reference pellet exhibited the lowest emission of this compound, averaging 88.71 mg∙m^−3^. This outcome may be associated with the high content of volatile components and the presence of tar-like substances in the bio-oil, which promote incomplete combustion. Additionally, the highest NOx emissions (1143.58 mg∙m^−3^) were also observed for pellets with the addition of bio-oil from dry tomato haulms (ad.1), which likely results from the increased nitrogen content in the fuel. Under high-temperature conditions, nitrogen compounds present in the biomass are oxidized, leading to the formation of nitrogen oxides. The lowest NOx emission was recorded for the reference pellet, which had the lowest nitrogen content (0.08%) and the lowest combustion temperature. These factors reduce NOx formation both through fuel-bound mechanisms and thermal reaction pathways.

### 3.4. Results of the Analysis of Suspended Dust Content in Exhaust Gases

**Figure 6:**
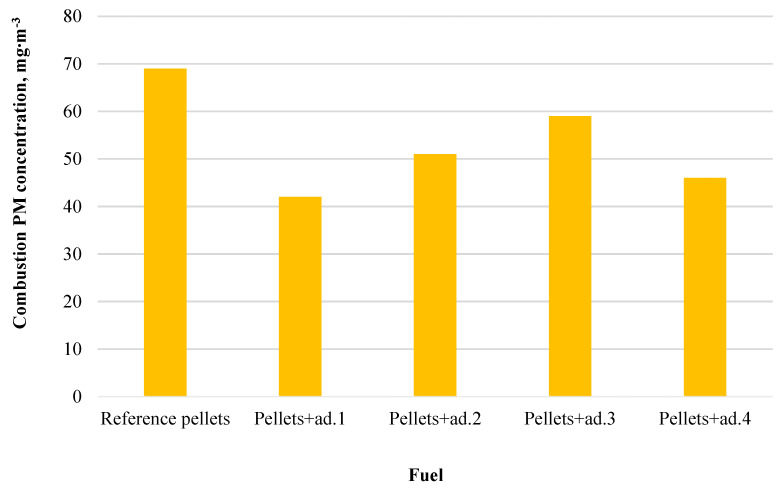
The content of suspended dust in the exhaust gases.

The chart below (Figure 6) shows the emission level of suspended dust (PM) during the combustion of pellets with the addition of bio-oil. 

The highest dust emissivity was observed in the case of the reference pellet (69 mg∙m^−3^), which can be associated with its lowest mechanical strength (96.24%), resulting in fuel crumbling and an increased amount of small fractions carried away during combustion. The lowest dust emission was recorded for pellets with the addition of bio-oil from dry tomato haulms (ad.1, 42 mg∙m^−3^), which also demonstrated one of the highest mechanical strengths (99.03%) and the highest combustion temperature (699.67 °C), favoring the more complete combustion of solid particles. The remaining samples showed intermediate values, indicating a relationship between pellet strength, combustion intensity, and dust emission.

## 4. Discussion

The use of bio-oil as a binder in the production of molded fuels significantly influenced their mechanical and physicochemical properties. Noticeable differences between individual pellets result not only from the presence of the binder itself but also from the type of biomass used to produce it. This resulted in its chemical composition, molecular structure, and organic compound content.

The increase in the calorific value in all pellets with binders may be directly related to the hydrocarbon compounds present in the bio-oil, as well as its viscous structure, which facilitated the binding of wood dust particles and thus reduced the porosity of the material. The highest calorific values were achieved by pellets with the addition of bio-oil from tomato fruit and bio-waste. The increase in the calorific value of bio-oil pellets, especially in the case of bio-waste (23.77 MJ∙kg^−1^) and tomato fruit (21.02 MJ∙kg^−1^), may be due to the higher carbon content (55.00% and 50.00%, respectively) and lower moisture content in both fuels. A higher share of carbon compounds and a lower water content promoted more complete combustion and a higher calorific value.

The type of bio-oils used and the degree of their carbonization influenced the differences in the content of volatile parts obtained biofuels. The lower content of volatile matter in pellets with the addition of bio-oil from dry tomato haulms (6.57%) may be the result of the greater thermal stability of these oils, containing less low-molecular-weight hydrocarbons and aromatic compounds. The higher content of volatile parts in pellets with bio-oil from bio-waste (15.70%) and tomato fruit (14.71%) is probably due to the presence of a larger amount of light, reactive organic fractions, which is characteristic of bio-oils obtained in the process of the rapid pyrolysis of biomass [24].

The key impact of the use of binders is also visible in the mechanical strength of pellets. In addition to the binder function, the bio-oils also served as a plasticizer, increasing the bonds between wood dust particles. All modified samples achieved very high mechanical strength values (>98.6%), with the highest observed for ad.3 (99.11%), ad.2 (99.09%), and ad.1 (99.03%). The slightly lower value for ad.4 (98.65%) still meets the A1 quality standard but may result from differences in composition, viscosity, or homogeneity. The reference sample showed the lowest mechanical strength (96.24%).

Analyzing the above results, the addition of bio-oil also influenced the change in the average temperature in the combustion chamber. The highest temperature (699.67 °C) was recorded for pellets with the addition of bio-oil from dry tomato haulms (ad.1), which may be due to its high calorific value or the presence of flammable organic components. The lowest temperature (617.87 °C) was recorded for the reference pellet. The reference pellet was characterized by the lowest calorific value (18.79 MJ∙kg^−1^), the lowest carbon content (48.00%) and the highest humidity (9.92%), which limited the combustion efficiency.

The use of bio-oil as a binder influenced not only the physicochemical parameters of the fuel but also the emission of pollutants during its combustion. The highest CO and NOx concentrations were recorded for pellets with the addition of bio-oil from dry tomato haulms (ad.1), which correlates with the higher nitrogen and volatile matter content in the fuel and elevated combustion temperatures. The reference pellet had the lowest emissions of both CO (88.71 mg∙m^−3^) and NOx (892.38 mg∙m^−3^), which is consistent with its lower nitrogen content (0.08%) and combustion temperature.

The addition of bio-oil as a binder had a significant impact on the reduction in dust emissions during pellet combustion. The lowest dust emission (42 mg∙m^−3^) was observed for ad.1 (dry haulms), which also had the highest combustion temperature and excellent structural stability. The highest emission was observed for the reference pellet (69 mg∙m^−3^), likely due to its lower mechanical integrity and increased crumbling. The other bio-oil-modified samples showed intermediate dust levels.

The above results suggest that not all bio-oils have the same properties, and their effectiveness as a binder depends directly on their chemical composition, degree of decarbonization, presence of binders, and water content.

## 5. Conclusions

Based on the research conducted, it can be concluded that the use of bio-oils as a binder in the production of molded fuels opens new possibilities for the management of bio-waste and post-production residues that have not been used for energy purposes so far. By enriching the biomass with organic compounds derived from bio-oils, it is possible to increase the calorific value of the fuel while maintaining the same mass of the raw material. Improving mechanical properties reduces material losses during transport and storage, and reducing dust emissions during combustion has a positive impact on the environment. The simultaneous increase in energy efficiency and reduction in the negative impact on the atmosphere indicates the potential for the wide use of bio-oils in the production of new-generation biofuels. In particular, the use of pyrolysis oil allows the utilization of agricultural waste that is difficult to process, such as haulm and tomato fruit, as well as bio-waste from municipal and industrial sectors. The results confirmed that all types of bio-oil used improved the physicochemical properties of the fuel and reduced environmental emissions compared to the reference sample. Among them, pyrolysis oil from dry tomato haulms showed the highest combustion temperature and the lowest dust emissions, indicating high effectiveness in terms of energy and environmental performance. In terms of mechanical strength, the best result was obtained for pellets containing bio-oil from tomato fruit. Due to the large amount of these residues and the limited possibilities of their traditional use, developing methods of processing them into valuable energy products is an important element of the circular economy and the Zero Waste philosophy. This solution includes not only the effective recovery of raw materials but also reducing the amount of waste and increasing the share of renewable energy sources in the energy mix. Research results confirm that bio-oils can effectively replace conventional binders in pellet production, leading to more environmentally friendly and sustainable energy solutions. Nevertheless, the key to the successful use of bio-oil remains its source and chemical composition, which suggests the need for further research on the optimization of the pyrolysis process and the selection of raw materials. Due to the promising results, it is also worth extending the research to evaluate the effect of different binders on the properties of other formed fuels and the possibility of obtaining binders from alternative waste materials. An important direction of future work may also be to determine the impact of the use of bio-oils on energy consumption in the pelletization process. In subsequent research cycles, the team will also create a model optimizing the dose and type of binder to obtain fuel of the highest possible quality and the lowest possible emissions resulting from its conversion.

## Figures and Tables

**Table 1:** Technical specification of Biowarmer 60 pellet boiler.

Parameter	Unit	Value
Boiler class according to the eco-design directive	-	5
Boiler output	kW	60
Boiler efficiency	%	87.3
Boiler weight	kg	420
Maximum operating temperature	°C	90
Minimum return water temperature	°C	70
Highest working overpressure	bar	2

**Table 2:** Technical data of low-power pyrolysis reactor.

Parameter	Unit	Value
Capacity	dm^3^	0.1–0.3
Working temperature range	°C	20–300 ± 0.1
Working pressure range	bar	1–100
Reactor output	W	1000

**Table 3:** Technical specifications of the IKA C 200 calorimeter.

Parameter	Unit	Value
Maximum output energy	J	40,000
Temperature sensor resolution	°C	0.0001
Working pressure of oxygen	bar	40
Initial temperature settings	°C	18–25

**Table 4:** Technical specifications of the TGA 701 analyzer.

Parameter	Unit	Value
Sample mass	g	1
Number of samples	pcs	19 (+1 reference)
Precision	%	0.02
Temperature control range	°C	100–1000
Temperature control accuracy	°C	±2
Temperature control stability	°C	±2
Maximum ramp rate from ambient to 104 °C	°C‧min^−1^	15
Maximum ramp rate from 104 °C to 1000 °C	°C‧min^−1^	50
Gas pressure	bar	3.1 for air, 2.4 for nitrogen, 2.4 for oxygen
Minimum gas purity	%	99.9 for nitrogen, 99.5 for oxygen

**Table 5:** Technical data of the PerkinElmer CHNS/0 2400 apparatus.

Parameter	Unit	Value
Temperature range	°C	100–1100
Sample size	mg	0–500
Accuracy	%	≤0.3
Carbon Analytical Range	mg	0.001–3.6
Hydrogen Analytical Range	mg	0.001–1.0
Nitrogen Analytical Range	mg	0.001–6.0
Sulphur Analytical Range	mg	0.001–2.0
Oxygen Analytical Range	mg	0.001–2.0

**Table 6:** Results of elemental analysis of pellets.

Parameter	Unit	Reference Pellet	Pellets+ ad.1	Pellets+ ad.2	Pellets+ ad.3	Pellets+ ad.4
Nitrogen content	%	0.08 ± 0.02	0.22 ± 0.03	0.35 ± 0.05	0.28 ± 0.03	0.56 ± 0.04
Carbon content	%	48.00 ± 0.1	53.00 ± 0.2	51.00 ± 0.1	50.00 ± 0.1	55.00 ± 0.1
Hydrogen content	%	8.20 ± 0.05	8.30 ± 0.05	8.40 ± 0.05	8.30 ± 0.05	8.50 ± 0.05
Sulfur content	%	0.017 ± 0.01	0.13 ± 0.02	0.18 ± 0.03	0.016 ± 0.01	0.11 ± 0.02

**Table 7:** Results of the technical analysis of pellets.

Parameter	Unit	Reference Pellet	Pellets+ ad.1	Pellets+ ad.2	Pellets+ ad.3	Pellets+ ad.4
Lower heating value	MJ∙kg^−1^	19.4 ± 0.1	22.6 ± 0.2	21.8 ± 0.15	21.0 ± 0.3	23.8 ± 0.2
Moisture content	%	8.4 ± 0.1	6.4 ± 0.2	6.6 ± 0.1	8.2 ± 0.15	6.5 ± 0.1
Volatile matter content	%	10.0 ± 0.1	6.6 ± 0.2	12.2 ± 0.2	14.7 ± 0.2	15.7 ± 0.1
Ash (mineral parts)	%	0.4 ± 0.05	0.8 ± 0.1	0.8 ± 0.05	0.7 ± 0.1	0.3 ± 0.05

## Data Availability

The original contributions presented in this study are included in the article. Further inquiries can be directed to the corresponding author.
